# Curcumin/cyclodextrin polymer inclusion complex attenuates ethanol‐induced liver injury by inhibition of DNA damage in mice

**DOI:** 10.1002/fsn3.3248

**Published:** 2023-02-02

**Authors:** Jianping Chen, Tugui Fan, Jiarui Li, Rui Li, Xiaofei Liu, Bing Wu, Jialong Gao, Ying Liu, Hao Dong, Saiyi Zhong

**Affiliations:** ^1^ College of Food Science and Technology Guangdong Ocean University, Guangdong Provincial Key Laboratory of Aquatic Product Processing and Safety, Guangdong Provincial Engineering Technology Research Center of Seafood, Guangdong Province Engineering Laboratory for Marine Biological Products, Key Laboratory of Advanced Processing of Aquatic Product of Guangdong Higher Education Institution Zhanjiang China; ^2^ Collaborative Innovation Center of Seafood Deep Processing Dalian Polytechnic University Dalian China; ^3^ College of Costal Agricultural Sciences Guangdong Ocean University Zhanjiang China; ^4^ College of Light Industry and Food Sciences Zhongkai University of Agriculture and Engineering Guangzhou China

## Abstract

This study was to examine the protective effects of curcumin/cyclodextrin polymer inclusion complex (CUR/CDP) on ethanol‐induced liver injury in mice and to explore its potential mechanisms. In the ethanol‐induced acute injury mouse model, the effects of pretreatment with silymarin, cyclodextrin polymer (CDP), curcumin (CUR) and CUR/CDP at low, middle, and high doses were evaluated by biochemical and histopathological examination. The liver index, alanine aminotransferase (ALT), aspartate aminotransferase (AST), and lactate dehydrogenase (LDH) levels in serum of the mice were measured. The superoxide dismutase (SOD), glutathione peroxidase (GSH‐PX) activities, and malondialdehyde (MDA) level in liver tissue were assessed by assay kits. Moreover, hematoxylin–eosin (HE) staining was carried out to observe pathological changes of liver. Western blotting was performed for determining the changes in the expressions of DNA damage‐associated proteins. The results showed that compared with the control group, the liver index and the levels of ALT, AST, LDH, and MDA in the ethanol treatment group were significantly increased and the activities of GSH‐Px and SOD were obviously decreased. However, pretreatment with silymarin, CUR, and CUR/CDP reversed the change of above indicators except CDP. Moreover, CUR/CDP at high dose further weakened the liver index, inhibited the biochemical indexes, and enhanced the activities of antioxidant enzymes to a greater extent than silymarin and CUR. Western blot analysis indicated that CUR/CDP significantly down‐regulated the expressions of DNA damage‐related proteins including p‐ATM, γ‐H2AX, p‐p53, and p‐p38MAPK, which inhibited ethanol‐induced the G2/M arrest and ultimately prevented liver function from oxidative stress injury. These results indicated that CUR/CDP possessed good protective effect on mice liver damage in vivo by increasing the activities of GSH‐Px and SOD to suppress DNA damage.

## Graphical abstract

CUR/CDP exhibited excellent protective effect on ethanol‐induced liver injury in mice in a dose‐dependent manner, which was attributed to its antioxidant activity that alleviated oxidative stress by improvement activities of antioxidant enzymes and inhibition of lipid peroxidation, thereby avoiding DNA damage‐induced G2/M arrest. CUR/CDP had the potential to be used as a novel drug or supplement for the prevention of alcoholic liver injury.

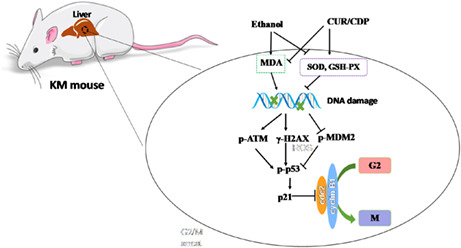

## 1 INTRODUCTION

Alcoholic liver disease (ALD) is a disorder caused by prolonged high alcohol intake, which can progress to fatty liver, liver fibrosis, and even liver cirrhosis and hepatocellular carcinoma (Samuhasaneeto et al., 2009). Although the pathogenesis of ALD is not clear, the oxidative metabolites of ethanol, such as acetaldehyde and reactive oxygen species (ROS) play an important role in the occurrence and development of ALD (Yuan et al., 2018). Overproduction of ROS results in oxidative stress that initiates cell arrest and cell injury (Chen et al., 2022; Dai et al., 2019). Therefore, finding an effective natural compound for the protection of liver from oxidative stress and cell injury would be beneficial for preventing liver diseases.

In recent years, plant phytochemicals have been found to possess hepatoprotective activities (Cui et al., 2014; Wang et al., 2014). Curcumin (CUR), a polyphenolic compound, is isolated from the rhizomes of *Curcuma longa* (Uzunhisarcikli & Aslanturk, 2019). Numerous studies have also shown that CUR possesses antioxidant and hepatoprotective activities in vivo (Hashish & Elgam, 2006; Palipoch et al., 2014). However, its application is limited because of its hydrophobicity. β‐cyclodextrin (β‐CD) containing seven glucopyranose units has been widely used to improve drug's bioavailability and solubility due to its reasonable cavity size (Huang et al., 2013). However, its low aqueous solubility limited its application in formulations (Gidwani & Vyas, 2014). To solve this problem, hydrophilic derivatives of β‐CD like β‐cyclodextrin polymer (CDP) have been prepared and found that CDP could increase the solubility and spectral properties of the poorly water‐soluble drugs (Zhang et al., 2013). Therefore, in our previous study, CUR was modified with β‐cyclodextrin polymer (CDP) to form curcumin/β‐cyclodextrin polymer inclusion complex (CUR/CDP), which improved curcumin's solubility (Chen et al., 2018). Yet, little is known about the detailed effects of CUR/CDP on alcoholic liver injury.

Therefore, in the present study, the protective effect of curcumin/cyclodextrin polymer inclusion complex (CUR/CDP) on ethanol‐induced liver injury in mice was investigated and its potential mechanism was explored.

## 2 MATERIALS AND METHODS

### 2.1 Materials

CUR/CDP (containing 10.78% CUR) was prepared in our laboratory (Chen et al., 2018). Curcumin was purchased from Sigma Chemical Co. A bicinchoninic acid (BCA) kit was obtained from Beyotime Biotechnology. Assay kits for total superoxide dismutase (SOD), glutathione peroxidase (GSH‐PX), malondialdehyde (MDA), aspartate aminotransferase (AST), alanine aminotransferase (ALT), and lactate dehydrogenase (LDH) were purchased from Nanjing Jiancheng Biology Engineering Institute (Nanjing, Jiangsu, China). Primary antibodies against cyclin B1, p‐ATM, p53, and γ‐H2AX were purchased from Santa Cruz Biotechnology. Primary antibodies against phospho‐p53 (Ser15) and p21 were obtained from Cell Signaling Technology and Abcam Company, respectively. Other chemical reagents were purchased from China National Pharmaceutical Group Co., Ltd.

### 2.2 Animals

**FIGURE 1:**
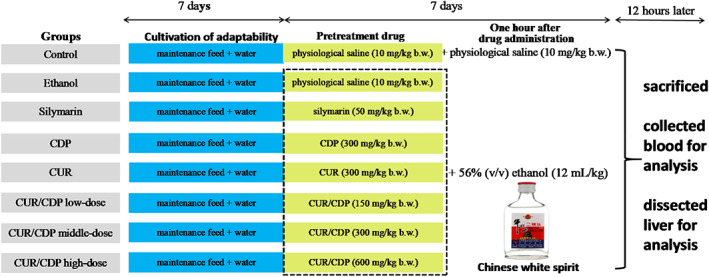
Experimental grouping and protocol.

Male Kunming (KM) mice were purchased from Beijing HFK Biotechnology Co., Ltd. The animal experiments were carried out following the guidelines of the Guangdong Ocean University Ethics Committee (IACUC No.: GDOU‐LAE‐2020‐007). The experimental design was shown in Figure 1. Firstly, 80 KM mice (1.8–1.84 kg) received maintenance feed and drank water for 7 days. Then, the mice were randomly divided into eight groups (10 mice per group): control group, ethanol group, silymarin group, CDP group, CUR group, CUR/CDP low‐dose group, CUR/CDP middle‐dose group, and CUR/CDP high‐dose group. For the control and ethanol groups, the mice were orally administered 10 ml/kg b.w. physiological saline daily for the next 7 days. For the silymarin group, the mice were orally administered 50 mg/kg b.w. silymarin daily for the next 7 days. For the CDP group, the mice were orally administered 300 mg/kg b.w. CDP daily for the next 7 days. For the CUR group, the mice were orally administered 300 mg/kg b.w. CUR daily for the next 7 days. For the CUR/CDP low‐dose group, the mice were orally administered 150 mg/kg b.w. CUR/CDP daily for the next 7 days. For the CUR/CDP middle‐dose group, the mice were orally administered 300 mg/kg b.w. CUR/CDP daily for the next 7 days. For the CUR/CDP high‐dose group, the mice were orally administered 600 mg/kg b.w. CUR/CDP daily for the next 7 days. One hour after drug administration, each group except for the control group was administered with 56% (v/v) ethanol (white spirit) at a dose of 12 ml/kg, while the control group received 10 ml/kg b.w. physiological saline instead of alcohol. On day 14th, the blood were taken from the eyeballs of all mice under anesthesia and then the mice were sacrificed by cervical dislocation. All liver tissues were dissected and weighed. The liver index was calculated according to the following formula:

$$\text{Liver index}\;\left(\%\right)=\text{liver weight}/\text{body weight of the}\;\mathrm{rat}\;\mathrm{on}\;\text{sacrifice}\;\mathrm{day}\times 100\%.$$

### 2.3 Determination of serum AST, ALT, and LDH levels

The blood samples were centrifuged at 3000 *g* for 10 min, and the serum was collected. The activities of ALT, AST, and LDH in the serum were determined using respective assay kits according to the manufacturer's protocols.

### 2.4 Detection of SOD, GSH‐PX, and MDA

The equivalent part of the liver tissue from each mouse was accurately weighed and then homogenized with normal saline in an ice‐water bath according to a mass/volume ratio of 1:9. Following centrifugation at 3000 *g* for 15 min, the supernatant was collected and the activities of SOD and GSH‐PX and content of MDA in the liver homogenate were measured using respective kits according to the manufacturer's instructions.

### 2.5 Histopathological observation of liver tissue

The equivalent part of the liver tissue from each mouse was fixed with 4% paraformaldehyde solution and then paraffin‐embedded sections were made. The sections were stained with hematoxylin–eosin and observed under a light microscope.

### 2.6 Western blot analysis

Whole protein was extracted from liver tissue homogenate by RIPA lysis buffer. The protein concentration was determined using the BCA kit. Equal amounts of individual protein samples were separated by electrophoretic in SDS‐PAGE. After electrophoresis, proteins were transferred to a PVDF membrane and blocked with 5% non‐fat milk for 2 h. After incubation with primary antibodies at a dilution of 1:1000 overnight, the membranes were washed with TBST. The membranes were then incubated with secondary HRP‐labeled antibody for 2 h. Protein bands were visualized using a chemiluminescence reagent (ECL). The bands were calculated using Image J software.

### 2.7 Statistics and analysis

Each experiment was repeated three times and the experimental data are expressed as the mean ± standard deviation of the triplicate measurements. SPSS 18.0 software was used for statistical analysis of the experimental results, which were analyzed by one‐way ANOVA. The SNK method was used for pairwise comparison between groups, with differences considered significant at *α* = .05 (Li et al., 2019; Zhang et al., 2021).

## 3 RESULTS AND DISCUSSION

### 3.1 Effect of CUR/CDP on the liver index of mice

**FIGURE 2:**
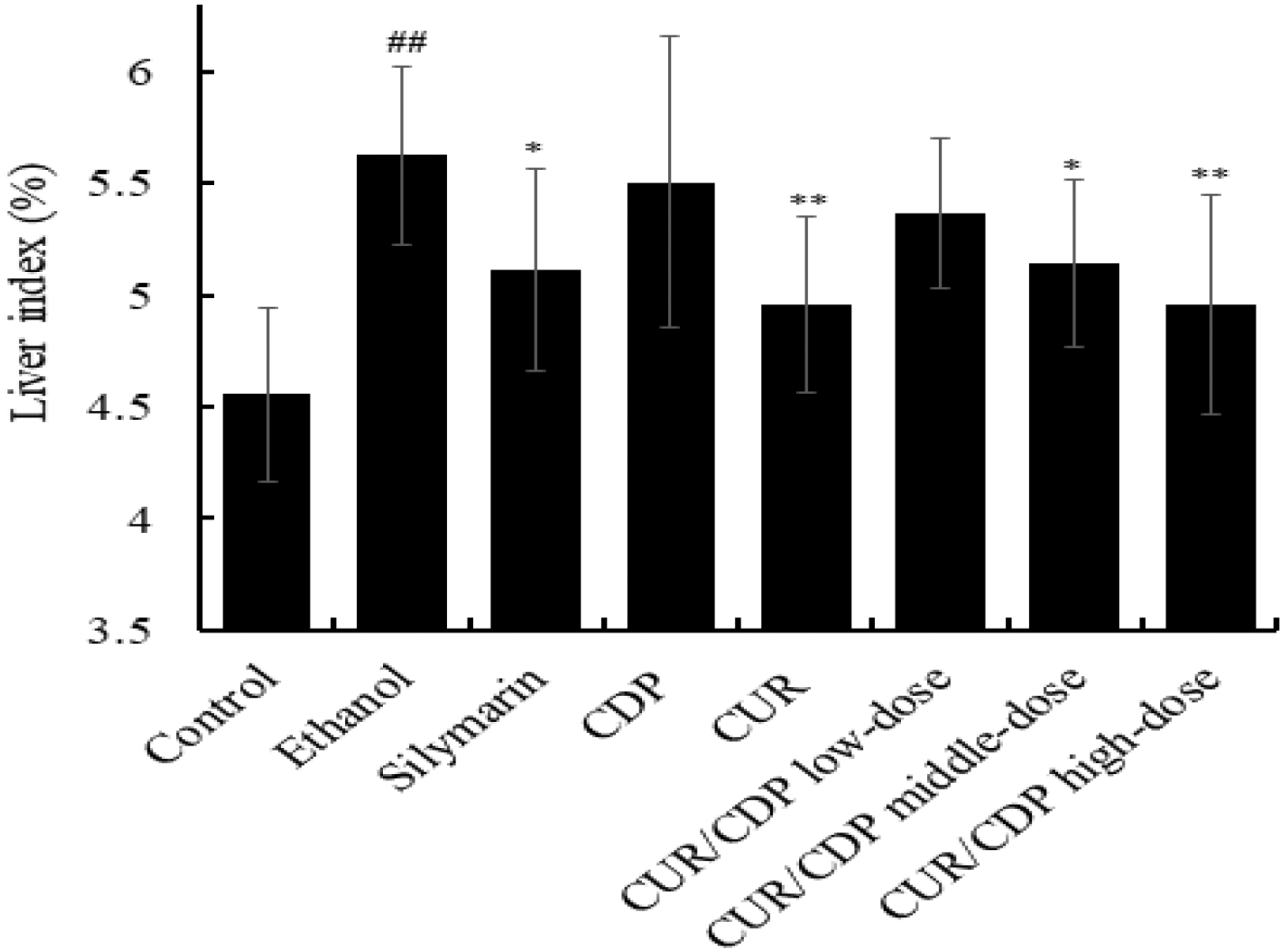
Effect of CUR/CDP on the liver index of mice. *p* < .01 (##) indicates significant differences between the control group and the ethanol group. *p* < .01 (**) and *p* < .05 (*) indicate significant differences between the ethanol group and other treatment groups.

Previous studies demonstrated that the liver index could reflect the level of liver damage to some extent (Hou et al., 2019). Hence, we firstly investigated the effect of CUR/CDP on the liver index. As shown in Figure 2, the liver index of the ethanol group increased from 4.55 ± 0.39% (control group) to 5.62 ± 0.40%, indicating that ethanol induced the liver injury in mice. However, compared with the ethanol group, pretreatment with silymarin, CDP, CUR, and CUR/CDP reduced the liver index. Moreover, CUR/CDP at high dose showed the same effect as CUR, which was greater than silymarin. These results showed that CUR/CDP could significantly reduce the liver index to improve alcohol‐induced liver injury in mice.

### 3.2 Effects of CUR/CDP on the activities of ALT, AST, and LDH in mice serum

**FIGURE 3:**
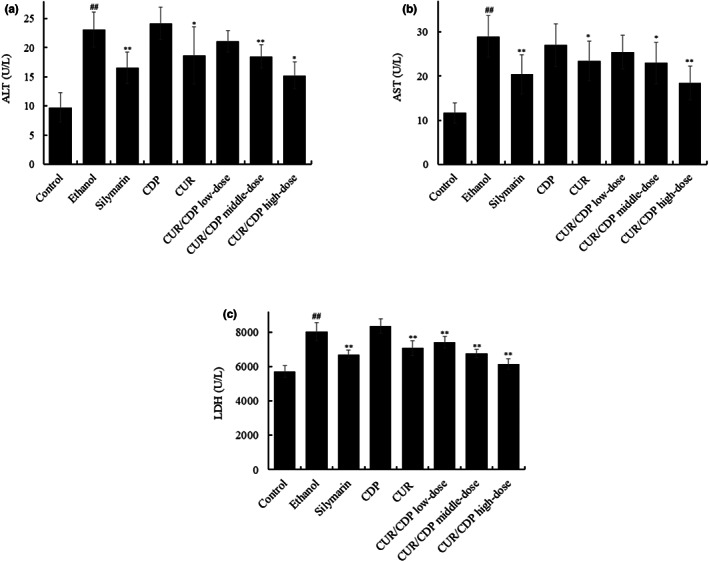
Effects of CUR/CDP on the activities of ALT (a), AST (b), and LDH (c) in mouse serum. After the mice were treated according to the procedure in Figure 1a, the ALT, AST, and LDH activities in serum were determined. *p* < .01 (##) indicates significant differences between the control group and the ethanol group. *p* < .01 (**) and *p* < .05 (*) indicate significant differences between the ethanol group and other treatment groups.

Under normal circumstances, ALT, AST, and LDH mainly exist in liver cells. When liver cells are injured, ALT, AST, and LDH can be released into the serum, which significantly increases the contents of ALT, AST, and LDH in serum. Therefore, the detection of ALT, AST, and LDH in the serum of mice could reflect the degree of liver injury in mice (Kumar et al., 1994; Reichling & Kaplan, 1988). In the present study, to further evaluate the degree of liver injury, we detected ALT, AST, and LDH levels using the commercial assay kits. As shown in Figure 3, compared with the control group, the activities of ALT, AST, and LDH were significantly increased in the ethanol group. However, silymarin, CUR, and CUR/CDP also significantly resulted in a decrease in the serum ALT, AST, and LDH levels compared with the ethanol group except CDP. Moreover, the ameliorative effect of CUR/CDP at high dose was more pronounced than CUR or silymarin. These results indicated that CUR/CDP could alleviate ethanol‐induced liver injury in mice by decreasing ALT, AST, and LDH activities.

### 3.3 Effects of CUR/CDP on SOD activity, GSH‐PX activity, and MDA content in mice liver

**FIGURE 4:**
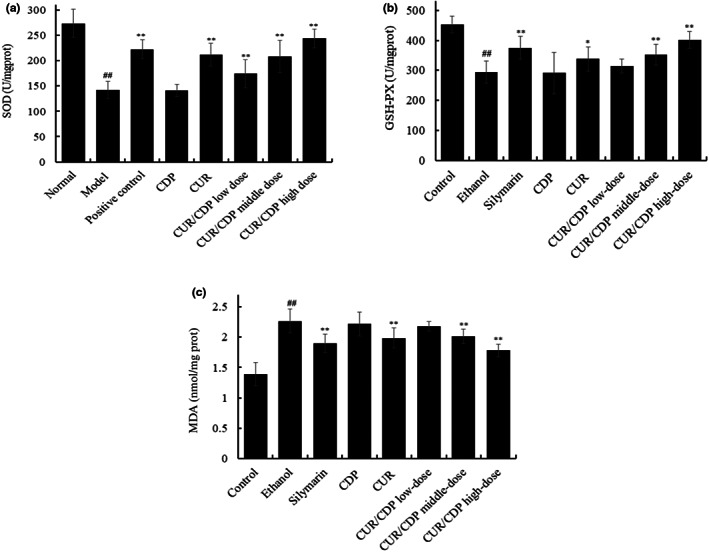
Effects of CUR/CDP on SOD activity (a), GSH‐PX activity (b), and MDA content (c) in mouse livers. After the mice were treated according to the procedure in Figure 1a, the SOD and GSH‐PX activities and MDA content in the liver were determined. *p* < .01 (##) indicates significant differences between the control group and the ethanol group. *p* < .01 (**) and *p* < .05 (*) indicate significant differences between the ethanol group and other treatment groups.

MDA is the main product of the intracellular lipid oxidation reaction and the content of MDA in cells reflects the degree of intracellular lipid peroxidation (Xia et al., 2017). GSH‐PX is a selenoenzyme that can catalyze the decomposition of hydrogen peroxide to water, thereby preventing cell membrane against oxidative damage (Xu et al., 2017). As the primary defense against the oxidation reaction, SOD can convert superoxide anion to hydrogen peroxide, thereby removing oxygen‐free radicals and prevent the body from damage (Wang et al., 2020; Xu et al., 2010). Therefore, when cells suffer from injured, GSH‐PX and SOD in cells can maintain redox homeostasis by scavenging oxygen‐free radicals in cells, thereby protecting cells. To investigate the effect of CUR/CDP on ethanol‐induced oxidative stress, the activities of SOD and GSH‐PX and the content of MDA were measured by the commercial assay kits. As shown in Figure 4, compared with the control group, significant decreases in the activities of SOD and GSH‐PX and a marked increase in the MDA content were observed in the ethanol group. However, silymarin, CUR, and CUR/CDP increased the activities of SOD and GSH‐PX and decreased the MDA content, while CDP did not significantly change the activities of SOD, GSH‐PX and the MDA content, indicating that silymarin, CUR and CUR/CDP had certain protective effects on the alcoholic liver injury in mice except CDP. Moreover, compared with CUR or silymarin, the level of MDA tended to decrease in the CUR/CDP high‐dose group, while the activities of SOD and GSH‐PX tended to increase. The results indicated that the protective effect of CUR/CDP high‐dose group on alcoholic liver injury was stronger than the CUR or silymarin group.

### 3.4 Effect of CUR/CDP on liver histopathology in mice

**FIGURE 5:**
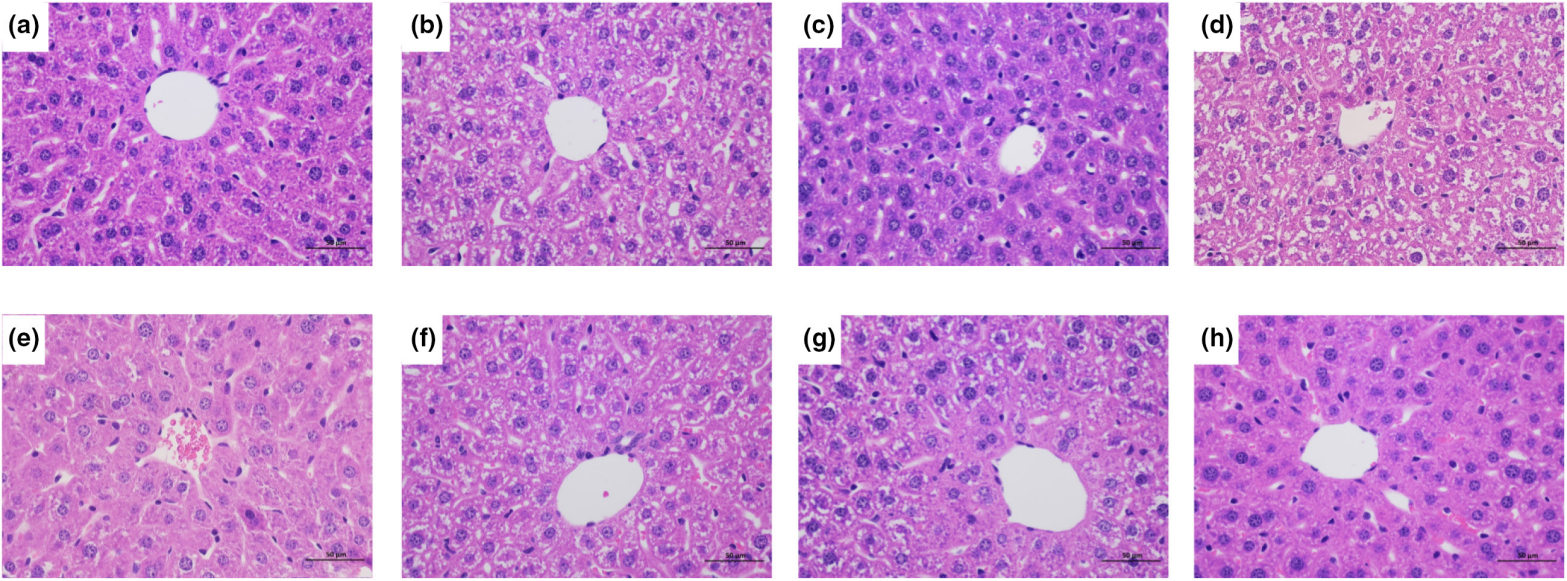
Observation of the effect of CUR/CDP on liver histopathology in mice (400×). (a) control group; (b) ethanol group; (c) silymarin group; (d) CDP group; (e) Cur group; (f) CUR/CDP low‐dose group; (g) CUR/CDP middle dose; (h) CUR/CDP high dose. After the mice were treated according to the procedure in Figure 1a, the pathological characteristics of liver tissue in mice were detected by HE staining and optical microscopy (scale bar = 50 μm).

Excessive intake of alcohol can cause liver tissue damage and pathological changes in mice (Jiang et al., 2020). Therefore, to evaluate the protective effect of CUR/CDP on ethanol‐induced liver damage, the histopathological changes in mice were examined by HE staining. As shown in Figure 5, ethanol caused disordered hepatic cords. There were no obvious boundaries between liver cells and the nuclei were located on one side of the liver cells. The cytoplasm of the liver cells was loose and there were many fat vacuoles of different sizes in the cytoplasm. However, after administration with CUR/CDP at 300 and 600 mg/kg, the cytoplasm of hepatocytes was more uniform. Moreover, CUR/CDP at 300 and 600 mg/kg remarkably improved the disorder of hepatic cord and the fat vacuoles in cytoplasm, which were similar to that of the silymarin group, and the improvement effects were better than that of the CUR group and the CDP group. These experimental results showed that CUR/CDP possessed a certain protective effect on ethanol‐induced liver injury in mice.

### 3.5 Effects of CUR/CDP on ethanol‐induced DNA damage and its related regulatory protein levels

**FIGURE 6:**
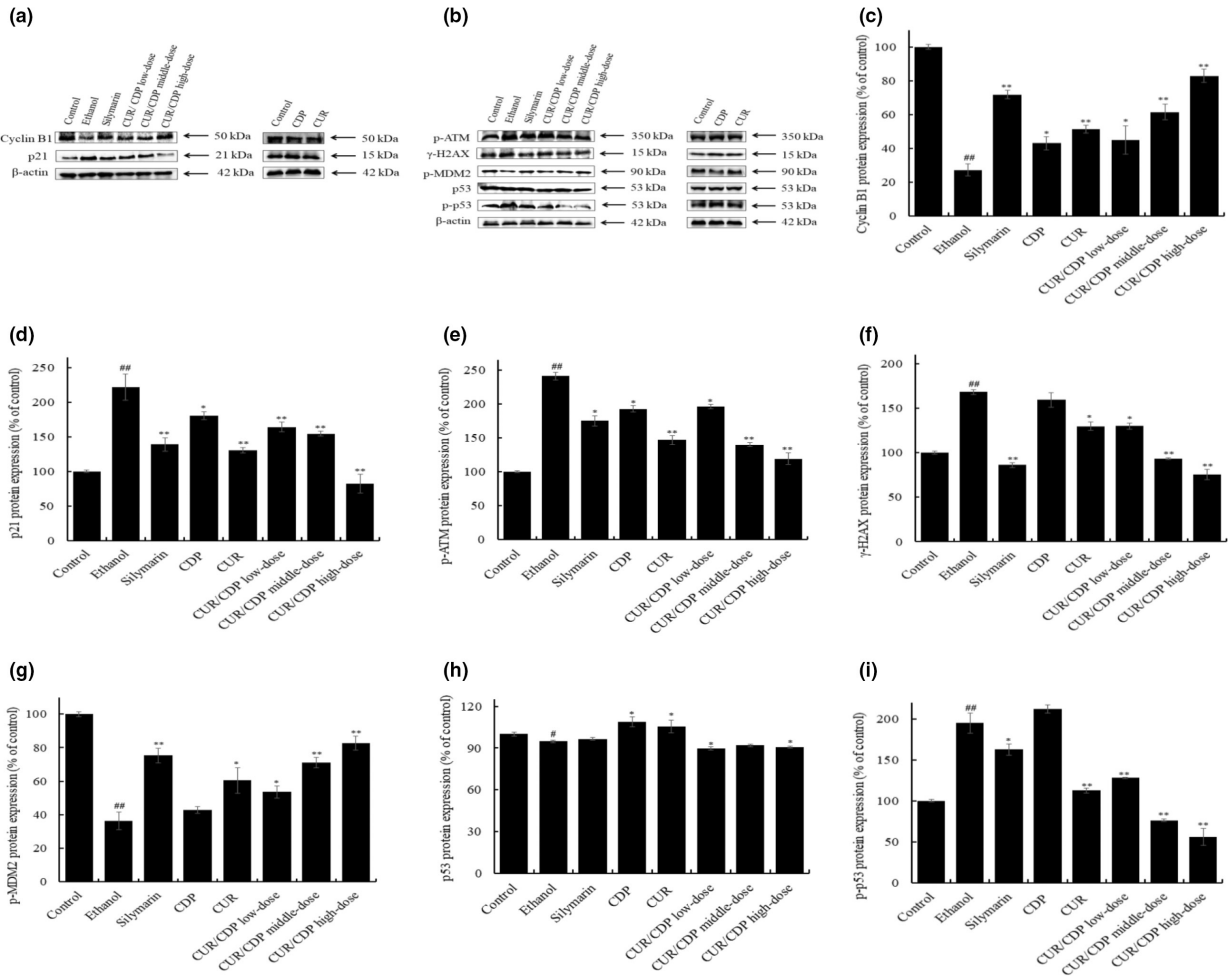
Effects of CUR/CDP on the expressions of cyclin and its upstream proteins in mice. (a) Protein bands of cyclin B1, p21, phosphorylated ATM, and γ‐H2AX. After the mice were intragastrically treated with different dosages of CUR/CDP at 150 mg/kg (low dose), 300 mg/kg (middle dose), or 600 mg/kg (high dose) for 7 days, liver tissue proteins were extracted and protein expression levels were detected by western blotting assay. (b) Protein bands of p‐MDM2, p53, and phosphorylated p53. After the mice were treated as described above, liver tissue proteins were isolated and protein expression levels were measured by western blotting analysis. Protein expression levels as percentages of control levels for (c) cyclin B1, (d) p21, (e) phosphorylated ATM, (f) γ‐H2AX, (g) phosphorylated MDM2, (h) p53, and (i) phosphorylated p53. *p* < .01 (##) and *p* < .05 (#) indicate significant differences between the control group and the ethanol group. *p* < .01 (**) and *p* < .05 (*) indicate significant differences between the ethanol group and other treatment groups.

Studies have shown that ethanol induces oxidative DNA damage in human peripheral lymphocytes (Min et al., 2018). Moreover, DNA damage could result in G2/M cell cycle arrest. It is reported that cyclin B1 and its kinase cdc2 (CDK1) play an important role in the regulation of G2/M phase (Kawamoto et al., 1997). Moreover, p21, a G_2_/M phase CDK inhibitor, accelerates G2/M arrest through binding to CDK1 leading to cyclinB1/cdc2 activity inhibition (Cheng et al., 2009). Therefore, to investigate whether ethanol‐induced DNA damage was related to G2/M arrest, we detected the expression of cyclin and its upstream proteins by western blotting analysis. As shown in Figures 6a,e,f, compared with the control group, the decrease in cyclin B1 expression and the increase in p21 expression were observed in ethanol‐treated groups, indicating that ethanol has induced G_2_/M arrest. However, compared with the ethanol group, CUR/CDP at 150, 300, and 600 mg/kg obviously increased cyclin B1 protein expression from 27.19% (ethanol group) to 44.91%, 61.47% and 82.91% and decreased p21 protein expression from 221.7% (ethanol group) to 163.83%, 154.52%, and 82.23%, respectively. In addition, p21 expression in the CUR/CDP at high‐dose group was lower than that of CUR and CDP groups, and cyclin B1 expression in the CUR/CDP at the high‐dose group was higher than that of CUR and CDP groups. These results indicated that compared with CUR and CDP groups, CUR/CDP showed stronger inhibition effect on ethanol‐induced G2/M arrest.

Studies have found that p53 plays a vital role in inducing cell cycle arrest and it can increase the p21 protein expression in response to DNA damage (Tewari et al., 2020). γ‐H2AX is considered to be one of the specific markers of DNA damage in tissues and cells (Gao et al., 2016; Stewart & Pietenpol, 2001). DNA damage could increase the phosphorylation of ATM (ataxia telangiectasia mutated) protein to induce apoptosis or arrest (Ohba et al., 2020). Meanwhile, DNA damage also could reduce the content of MDM2, leading to p53 inactivation (Yu et al., 2016). Therefore, to further confirm that DNA damage was involved in the process of alcoholic liver injury, we detected the expressions of phosphorylated ATM, γ‐H2AX, p53, phosphorylated p53, and phosphorylated MDM2. As shown in Figure 6c,g–k, compared with the control group, ethanol increased the expression levels of phosphorylated ATM, γ‐H2AX and phosphorylated p53 and decreased the expression level of phosphorylated MDM2, indicating that alcohol treatment caused DNA damage in the liver tissue. However, CUR/CDP treatment at different doses reversed the expressions of the above proteins. Moreover, phosphorylated ATM, γ‐H2AX, and phosphorylated p53 expressions in the CUR/CDP at the high‐dose group were lower than that of CUR and CDP groups, and phosphorylated MDM2 expression in the CUR/CDP at high‐dose group was higher than that of CUR and CDP groups. These results indicated that compared with CUR and CDP groups, CUR/CDP showed stronger inhibition effect on DNA damage.

### 3.6 Diagram of signaling pathway involved in inhibition of ethanol‐induced liver injury in mice by CUR/CDP

**FIGURE 7:**
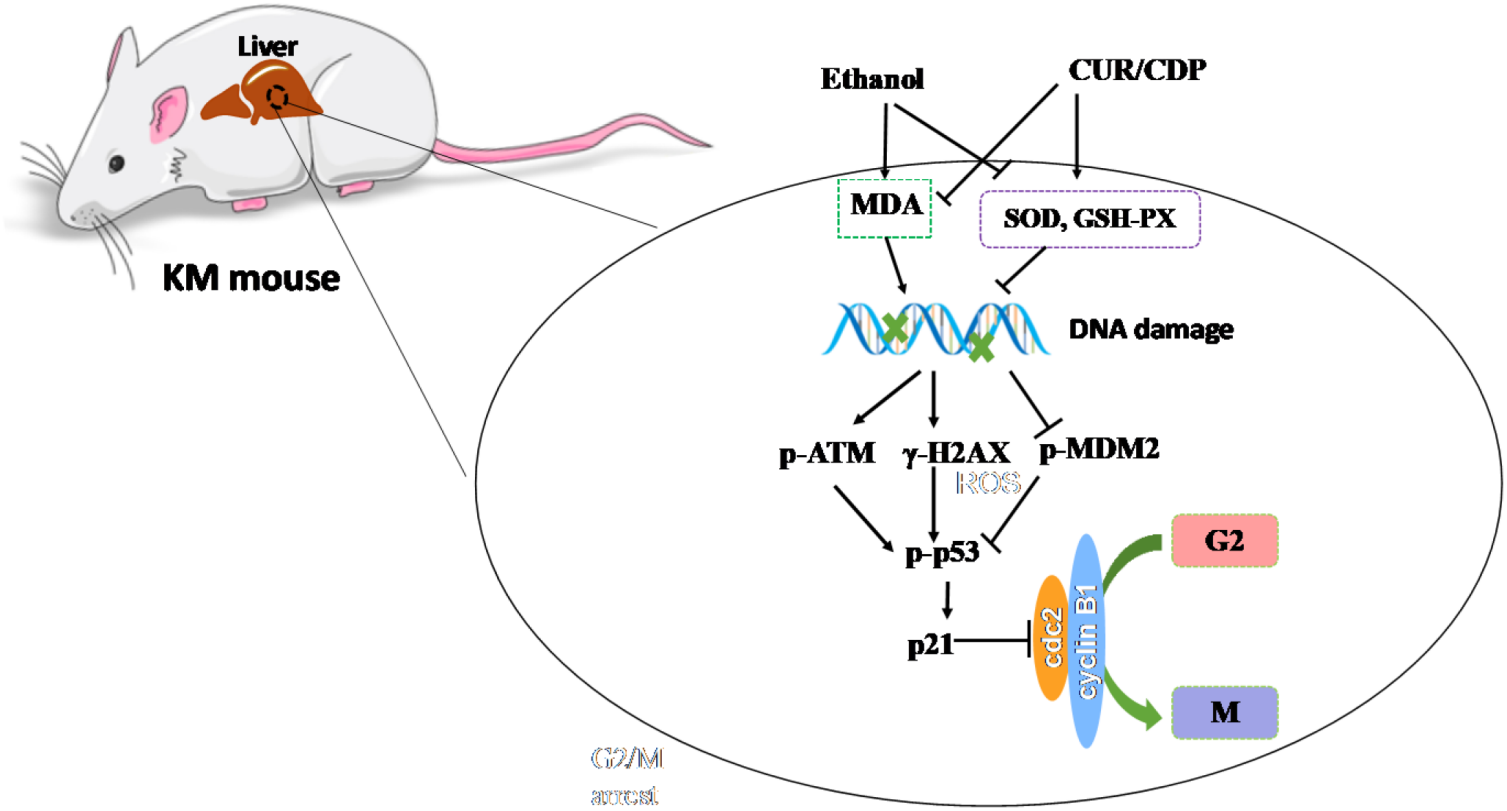
Possible mechanism of CUR/CDP alleviating ethanol‐induced hepatocyte injury in mice.

In this study, a signal pathway diagram of the possible involvement of CUR/CDP in inhibiting ethanol‐induced liver injury in mice was established. As shown in Figure 7, ethanol treatment resulted in decreases in the activities of SOD and GSH‐Px and an increase in the MDA content, thereby leading to oxidation stress. Subsequently, oxidation stress causes DNA damage, thereby increasing the expression levels of γ‐H2AX and p‐ATM and decreasing the expression levels of p‐MDM2 content. The increases of γ‐H2AX and p‐ATM promoted the phosphorylation of p53 protein, which increased the expression of p21 protein. The increase in p21 protein reduced the activity of CyclinB1‐cdc2 complex, thereby causing G2/M arrest. After treatment with CUR/CDP, CUR/CDP significantly increased the activity of SOD and GSH‐Px and decreased the MDA content, thus attenuating DNA damage‐induced G2/M arrest.

## 4 CONCLUSION

In conclusion, CUR/CDP exhibited excellent protective effect on ethanol‐induced liver injury in mice in a dose‐dependent manner, which was attributed to its antioxidant activity that alleviated oxidative stress by improvement activities of antioxidant enzymes and inhibition of lipid peroxidation, thereby avoiding DNA damage‐induced G2/M arrest. These results indicated that CUR/CDP had the potential to be used as a novel drug or supplement for the prevention of alcoholic liver injury.

## FUNDING INFORMATION

This work was funded by the Guangdong Basic and Applied Basic Research Foundation (2020A1515010860 and 2021A1515012455), Guangdong Ocean University Innovation Program (230419100), Nanhai Youth Scholar Project of Guangdong Ocean University (002029002009), the Project of Science and Technology of Zhanjiang City (2020A01034 and 2022A01045), the Scientific Research Foundation of Guangdong Ocean University (no. R17034), the Innovative Team Program of High Education of Guangdong Province (2021KCXTD021), Excellent Graduate Thesis Cultivation Program (040505042203; 040505042204) and Guangdong Provincial Key R&D Program (2022B0202040001).

## CONFLICT OF INTEREST

There are no conflicts to declare.

## Data Availability

Not applicable.
